# A numerically stable algorithm for integrating Bayesian models using Markov melding

**DOI:** 10.1007/s11222-022-10086-2

**Published:** 2022-02-18

**Authors:** Andrew A. Manderson, Robert J. B. Goudie

**Affiliations:** 1 MRC Biostatistics Unit, Forvie Site, Robinson Way, Cambridge, CB2 0SR UK; 2 The Alan Turing Institute, British Library, London, UK

**Keywords:** Biased sampling, Data integration, Evidence synthesis, Kernel density estimation, Multi-source inference, Self-density ratio, Weighted sampling

## Abstract

When statistical analyses consider multiple data sources, Markov melding provides a method for combining the source-specific Bayesian models. Markov melding joins together submodels that have a common quantity. One challenge is that the prior for this quantity can be implicit, and its prior density must be estimated. We show that error in this density estimate makes the two-stage Markov chain Monte Carlo sampler employed by Markov melding unstable and unreliable. We propose a robust two-stage algorithm that estimates the required prior marginal self-density ratios using weighted samples, dramatically improving accuracy in the tails of the distribution. The stabilised version of the algorithm is pragmatic and provides reliable inference. We demonstrate our approach using an evidence synthesis for inferring HIV prevalence, and an evidence synthesis of A/H1N1 influenza.

## Introduction

Many modern applied statistical analyses consider several data sources, which differ in size and complexity. The wide variety of problems and information sources has produced numerous methods for multi-source inference (Lanckriet et al. 2004; Coley et al. 2017; Besbeas and Morgan 2019), as well as general methodologies including evidence synthesis methods (Sutton and Abrams 2001; Ades and Sutton 2006; Spiegelhalter et al. 2004), and the data fusion model (Kedem et al. 2017). These methods require an appropriate joint model for all data, which can be challenging to specify.

An alternative approach is to model smaller, simpler aspects of the data, such that designing these *submodels* is easier, then combine the submodels. The premise is that the combination of many smaller submodels will serve as a good approximation to a larger joint model, which may be methodologically or computationally infeasible. *Markov melding* (Goudie et al. 2019) is a methodology for coherently combining these submodels. Specifically, Markov melding joins together submodels that share a common quantity $\phi$ into a single joint model. Consider $M$ submodels indexed by $m= 1, \ldots , M$ that share $\phi$, have submodel specific parameters $\psi _{m}$ and submodel specific data $Y_{m}$, denoting the $m^{th}$ submodel $\text {p}_{m}(\phi , \psi _{m}, Y_{m})$. Markov melding forms a single joint *melded model* $\text {p}_{\text {meld}}(\phi , \psi _{1}, \ldots , \psi _{M}, Y_{1}, \ldots , Y_{M})$, which enables information to flow through $\phi$ from one model to another. The melded model posterior thus incorporates uncertainty from all sources of data.

Multi-stage sampling methods are useful, pragmatic tools for estimating complex joint models – such as $\text {p}_{\text {meld}}$ – in a computationally feasible manner, and have been applied in settings including statistical genetics and phylogeny (Tom et al. 2010), meta-analysis (Lunn et al. 2013; Blomstedt et al. 2019), spatial statistics (Hooten et al. 2019), and joint models in survival analysis (Mauff et al. 2020). Whilst it is preferable to sample the joint posterior directly, this is often infeasible due to the complexity of the model, the size of the data, the limitations of probabilistic programming languages such as JAGS and Stan, or the complications of re-expressing complicated submodels in a common programming language (Johnson et al. 2020). Improving the stability of multi-stage estimation techniques is thus of interest to applied statisticians.

Evidence synthesis models consider multiple sources of data (evidence), including randomised controlled trials or observational studies, to understand complex phenomena. Each source of data has an associated submodel and set of parameters it informs; combining all the submodels requires assuming deterministic or probabilistic relationships between the submodel-specific parameters. For example, De Angelis et al. (2014) collected many surveys of partially overlapping subpopulations, albeit at different frequencies, and combined these in an evidence synthesis model to estimate human immunodeficiency virus (HIV) prevalence in the United Kingdom. An introduction to evidence synthesis can be found in Chapter 8 of Spiegelhalter et al. (2004); other applications include estimating the prevalence of campylobacteriosis (Albert et al. 2011) and influenza (Presanis et al. 2014).

We can form evidence synthesis models by applying Markov melding to the various sources of data and their submodels. However, the common quantity $\phi$ may be a complex, non-invertible function of the parameters in one of the submodels. This is a challenge for Markov melding, as the method requires the prior marginal density of $\phi$ under each submodel $\text {p}_{m}(\phi )$, which may not be analytically tractable. Instead, prior samples of $\phi$ are drawn, and the prior marginal density is estimated using a kernel density estimate (KDE) ${\widehat{\text {p}}}_{m}(\phi )$ (Wand and Jones 1995). However, the use of a KDE in lieu of the analytic density function has poor implications for the numerical stability of the Markov Chain Monte Carlo (MCMC) method used to estimate the melded posterior, even in our low dimensional examples. Specifically, we illustrate that the multi-stage MCMC sampler of Goudie et al. (2019) is sensitive to error in ${\widehat{\text {p}}}_{m}(\phi )$, particularly in low probability regions.

To address this sensitivity, we first note that Markov melding strictly only requires an estimate of the *self-density ratio* (Hiraoka et al. 2014), $\text {r}(\phi _{\text {nu}}, \phi _{\text {de}}) = \text {p}_{m}(\phi _{\text {nu}}) \mathop {/} \text {p}_{m}(\phi _{\text {de}})$, as we will show in Sect. 2. In Sect. 3 we develop methodology that reduces the error in the self-density ratio estimate $\widehat{\text {r}}(\phi _{\text {nu}}, \phi _{\text {de}})$ by using weighted-sample KDEs (Vardi 1985; Jones 1991), which are more accurate in low probability regions. Multiple weighted-sample estimates of $\widehat{\text {r}}(\phi _{\text {nu}}, \phi _{\text {de}})$ are combined via a weighted average to further improve performance. We call this methodology *weighted-sample self-density ratio estimation* (WSRE), and demonstrate the effectiveness of our methodology in two examples. The first is a toy example from Ades and Cliffe (2002). We show that output from the multi-stage estimation process that uses WSRE is closer to reference samples than the *naive* approach, which uses a single KDE for ${\widehat{\text {p}}}_{m}(\phi )$. The second example is an involved evidence synthesis, previously considered in Goudie et al. (2019). Here we show that the multi-stage estimation process that employs WSRE produces plausible samples, whilst the naive approach produces nonsensical results. In these examples $\phi$ is a 1 or 2 dimensional quantity. We discuss the applicability of our method for higher dimensional $\phi$ in Sect. 6.

## Markov melding

The Markov melding framework is able to join together any number of submodels which share a common component $\phi$. As the examples in this paper only consider two submodels, we limit our exposition to the $M= 2$ model case; for the more general case see Goudie et al. (2019). Markov melding constructs a joint model using the conditional distributions for submodel-specific parameters $\psi _{m}$ and data $Y_{m}$, denoted $\text {p}_{m}(\psi _{m}, Y_{m} \mid \phi )$. These conditional distributions are then combined with a global prior for $\phi$ called the *pooled prior* $\text {p}_{\text {pool}}(\phi )$, which we discuss in Sect. 2.1. Mathematically, assuming that the supports of the relevant conditional, joint, and marginal distributions containing $\phi$ are appropriate, we define the *melded joint distribution* as

1 $$\begin{aligned} \text {p}_{\text {meld}}&(\phi , \psi _{1}, \psi _{2}, Y_{1}, Y_{2}) \nonumber \\&= \text {p}_{\text {pool}}(\phi )\text {p}_{1}(\psi _{1}, Y_{1} \mid \phi ) \text {p}_{2}(\psi _{2}, Y_{2} \mid \phi ) \end{aligned}$$

2 $$\begin{aligned}&= \text {p}_{\text {pool}}(\phi )\frac{\text {p}_{1}(\phi , \psi _{1}, Y_{1})}{\text {p}_{1}(\phi )} \frac{\text {p}_{2} (\phi , \psi _{2}, Y_{2})}{\text {p}_{2}(\phi )}. \end{aligned}$$

The submodel-specific conditional densities $\text {p}_{m}(\psi _{m}, Y_{m}) \mid \phi )$ may be analytically intractable. Hence, it is easier to work with the submodel-joint densities $\text {p}_{m}(\phi , \psi _{m}, Y_{m})$ and their prior marginal distributions $\text {p}_{m}(\phi )$ as specified in Eq. (2), because the former can be factorised into the data generating process specified during submodel construction.

### Forming the pooled prior

The pooled prior should represent previous knowledge of $\phi$ in the absence of other information. A general approach to constructing $\text {p}_{\text {pool}}(\phi )$ is to consider a weighted combination of the prior marginal distributions $\text {p}_{m}(\phi )$, with submodel weights $\lambda _{m}$. Selection of the pooling method and specific values of the weights is a topic covered in much detail elsewhere (Clemen and Winkler 1999; O’Hagan et al. 2006); a full summary of this field is beyond the scope of this article. For the examples considered in this paper we form $\text {p}_{\text {pool}}(\phi )$ via logarithmic pooling: $\text {p}_{\text {pool}}(\phi )\propto \text {p}_{1}(\phi )^{\lambda _{1}}\text {p}_{2}(\phi )^{\lambda _{2}}$, with $\lambda _{1} = \lambda _{2} = \frac{1}{2}$. Logarithmic pooling also allows us to use the methodology we develop in Sect. 3 in the pooled prior.

### Two-stage Markov chain Monte Carlo sampler

Directly estimating the melded model’s posterior distribution

3 $$\begin{aligned} \text {p}_{\text {meld}}&(\phi , \psi _{1}, \psi _{2} \mid Y_{1}, Y_{2}) \nonumber \\ \,&\propto \, \text {p}_{\text {pool}}(\phi )\frac{\text {p}_{1}(\phi , \psi _{1}, Y_{1})}{\text {p}_{1}(\phi )} \frac{\text {p}_{2} (\phi , \psi _{2}, Y_{2})}{\text {p}_{2}(\phi )}. \end{aligned}$$

necessitates simultaneously evaluating both submodels. This can be impractical if the submodels are implemented in different probabilistic programming languages or have bespoke implementations. We use the two-stage Markov chain Monte Carlo (MCMC) sampler of Goudie et al. (2019) to sample from $\text {p}_{\text {meld}}(\phi , \psi _{1}, \psi _{2} \mid Y_{1}, Y_{2})$ without the need to evaluate Eq. (3) all at once. This involves a two-stage MCMC procedure, first sampling from a partial product of the terms in Eq. (3), then using these samples as a proposal distribution in the second stage. The result is a convenient cancellation of the common terms in the stage two acceptance probability, whilst still ensuring that the final samples come from the melded posterior distribution of Eq. (3).

In stage one of the sampler we may, for example, opt to target the first submodel $\text {p}_{1}$, but with an (improper) flat prior for $\phi$

$$\begin{aligned} \text {p}_{\text {meld}, 1} (\phi , \psi _{1} \mid Y_{1}) \propto \frac{ \text {p}_{1}(\phi , \psi _{1}, Y_{1}) }{ \text {p}_{1}(\phi ) }, \end{aligned}$$

so we construct a standard Markov chain in which a proposed move from $(\phi , \psi _{1}) \rightarrow (\phi ^{*}, \psi _{1}^{*})$, with proposal density $\text {q}(\phi ^{*}, \psi _{1}^{*} \mid \phi , \psi _{1})$, is accepted with probability

4 $$\begin{aligned} \alpha&((\phi ^{*}, \psi _{1}^{*}), (\phi , \psi _{1})) \nonumber \\&= \frac{ \text {p}_{1}(\phi ^{*}, \psi _{1}^{*}, Y_{1}) \text {p}_{1} (\phi ) \text {q}(\phi , \psi _{1} \mid \phi ^{*}, \psi _{1}^{*}) }{ \text {p}_{1}(\phi , \psi _{1}, Y_{1}) \text {p}_{1} (\phi ^{*}) \text {q}(\phi ^{*}, \psi _{1}^{*} \mid \phi , \psi _{1}) }. \end{aligned}$$

This Markov chain asymptotically emits samples from $\text {p}_{\text {meld}, 1}$.

In stage two we update $\phi$ and $\psi _{2}$ using Metropolis-within-Gibbs updates, targeting the full melded posterior distribution of Eq. (3). Updating $\phi$ uses the stage one samples as a proposal distribution. For a sample of size $N$ from $\text {p}_{\text {meld}, 1}$ denoted $\{\phi _{n}^{(\text {meld}, 1)}\}_{n= 1}^{N}$ we sample an index $n^{*}$ uniformly at random between 1 and $N$, and use the corresponding value as the proposal $\phi ^{*} = \phi _{n^{*}}^{(\text {meld}, 1)}$. This results in a stage two acceptance probability for a move from $\phi \rightarrow \phi ^{*}$ of

5 $$\begin{aligned}&\begin{aligned} \alpha&(\phi ^{*}, \phi ) \nonumber \\&= \frac{ \text {p}_{\text {pool}} (\phi ^{*}) \text {p}_{1}(\phi ^{*}, \psi _{1}, Y_{1}) \text {p}_{2}(\phi ^{*}, \psi _{2}, Y_{2}) \text {p}_{1}(\phi ) \text {p}_{2}(\phi ) }{ \text {p}_{\text {pool}} (\phi ) \text {p}_{1}(\phi , \psi _{1}, Y_{1}) \text {p}_{2}(\phi , \psi _{2}, Y_{2}) \text {p}_{1}(\phi ^{*}) \text {p}_{2}(\phi ^{*}) } \\&\qquad \frac{ \text {p}_{1}(\phi , \psi _{1}, Y_{1}) \text {p}_{1}(\phi ^{*}) }{ \text {p}_{1}(\phi ^{*}, \psi _{1}, Y_{1}) \text {p}_{1}(\phi ) } \end{aligned} \nonumber \\&= \frac{ \text {p}_{\text {pool}} (\phi ^{*}) \text {p}_{2}(\phi ^{*}, \psi _{2}, Y_{2}) \text {p}_{2}(\phi ) }{ \text {p}_{\text {pool}} (\phi ) \text {p}_{2}(\phi , \psi _{2}, Y_{2}) \text {p}_{2}(\phi ^{*}) }, \end{aligned}$$

since all stage one terms cancel, providing a form of “modularisation” in the algorithm. The update for $\psi _{2}$ has an acceptance probability for a move from $\psi _{2} \rightarrow \psi _{2}^{*}$, drawn from a proposal distribution $\text {q}(\psi _{2}^{*} \mid \psi _{2})$, of

$$\begin{aligned} \alpha (\psi _{2}^{*}, \psi _{2}) = \frac{ \text {p}_{2} (\phi , \psi _{2}^{*}, Y_{2}) }{ \text {p}_{2} (\phi , \psi _{2}, Y_{2}) } \frac{ \text {q}(\psi _{2} \mid \psi _{2}^{*}) }{ \text {q}(\psi _{2}^{*} \mid \psi _{2}) }, \end{aligned}$$

as all terms that do not contain $\psi _{2}$ cancel. Samples from the melded posterior distribution for $\psi _{1}$, $\text {p}_{\text {meld}}(\psi _{1} \mid Y_{1}, Y_{2})$, can be obtained by storing the indices $n$ used to draw values of $\phi$ from $\{\phi _{n}^{(\text {meld}, 1)}\}_{n= 1}^{N}$ in stage two. The stored indices are then used to resample the stage one samples $\{\psi _{1, n}^{(\text {meld}, 1)}\}_{n = 1}^{N}$ yielding samples from $\text {p}_{\text {meld}}(\psi _{1} \mid Y_{1}, Y_{2})$.

An interesting property of Equations (4) and (5) is that our interaction with the unknown prior marginal distribution is limited to the *self-density ratio* $\text {r}(\phi , \phi ^{*}) = \text {p}_{m}(\phi ) \mathop {/} \text {p}_{m}(\phi ^{*})$. In Sect. 3 we develop methodology that uses self-density ratios to improve the numerical stability of the acceptance probability calculations.

We do not have to target $\text {p}_{\text {meld}, 1} (\phi , \psi _{1} \mid Y_{1})$ with an improper prior in stage one; we are free to choose any of the components of Eq. (3). The choice of stage one components will affect MCMC mixing, yet is often constrained by the practicalities of sampling the subposterior distributions. In the example of Sect. 5 the common quantity $\phi$ is a non-invertible function of parameters in $\text {p}_{1}$, and it is possible to sample from the subposterior $\text {p}_{1}(\phi , \psi _{1} \mid Y_{1})$ using JAGS. Hence, we draw stage one samples from $\text {p}_{1}(\phi , \psi _{1} \mid Y_{1})$, with stage two, implemented partially in Stan, accounting for the remaining terms: $1 \mathop {/} \text {p}_{1}(\phi )$, $\text {p}_{2}(\phi , \psi _{2} \mid Y_{2}) \mathop {/} \text {p}_{2}(\phi )$, and $\text {p}_{\text {pool}}(\phi )$. This process highlights another interesting advantage of Markov melding; we can use samples produced from one statistical software package in combination with a model implemented in another, mixing and matching as is most convenient.

### Naive prior marginal estimation

**Fig. 1:**
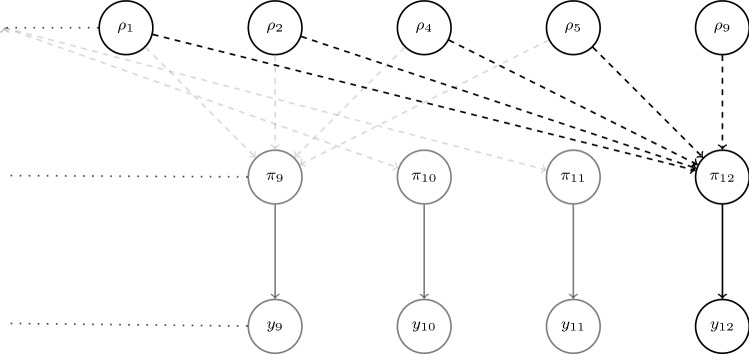
Partial directed acyclic graph (DAG) for the HIV model. The top row only depicts nodes that relate to $\pi _{12}$. Dashed lines indicate deterministic relationships between nodes, some of which are non-invertible. Solid lines indicate stochastic relationships

The expressions in Eqs. (4) and (5) explicitly include both models’ prior marginal distributions $\text {p}_{m}(\phi )$ for $m= 1, 2$, and implicitly includes them in $\text {p}_{\text {pool}}(\phi )$. In our examples we do not have analytic expressions for these marginals. More generally, if $\phi$ is not a root node in the directed acyclic graph representation of either submodel (see e.g. $\pi _{12}$ in Fig. 1), or is the aggregate output of a non-invertible deterministic link function, then the analytic form of $\text {p}_{m}(\phi )$ will likely be intractable.

The approach proposed by Goudie et al. (2019), which we call the *naive* approach, estimates the prior marginal distributions by sampling $\text {p}_{m}(\phi , \psi _{m}, Y_{m})$ for each model using simple Monte Carlo, as the samples of $\phi$ will be distributed according to the correct marginal, and employs a standard KDE ${\widehat{\text {p}}}_{m}(\phi )$ (Wand and Jones 1995). The two-stage sampler then targets the corresponding estimate of the melded posterior

6 $$\begin{aligned} {\hat{\text {p}}}_{\text {meld}}&(\phi , \psi _{1}, \psi _{2} \mid Y_{1}, Y_{2}) \nonumber \\ \,&\propto \, {\hat{\text {p}}}_{\text {pool}}(\phi ) \frac{\text {p}_{1}(\phi , \psi _{1}, Y_{1})}{{\hat{\text {p}}}_{1}(\phi )} \frac{\text {p}_{2} (\phi , \psi _{2}, Y_{2})}{{\hat{\text {p}}}_{2}(\phi )}, \end{aligned}$$

where ${{\hat{\text {p}}}}_{\text {pool}}(\phi )$ is the approximation to $\text {p}_{\text {pool}}(\phi )$ obtained by plugging in ${{\hat{\text {p}}}}_{m}(\phi )$ for $m= 1, 2$.

### Numerical issues in the naive approach

Sampling the melded posterior using Eq. (6) can be numerically unstable. Say we propose a move from $\phi \rightarrow \phi ^{*}$, where $\phi ^{*}$ is particularly improbable under $\text {p}_{m}$. The KDE estimate at this value, ${\widehat{\text {p}}}_{m}(\phi ^{*})$, is poor in terms of relative error

$$\begin{aligned} \left\| \frac{ \widehat{\text {p}}_{m} (\phi ^{*}) - \text {p}_{m} (\phi ^{*}) }{ \text {p}_{m} (\phi ^{*}) } \right\| _{1}, \end{aligned}$$

particularly in the tails of the distribution (Koekemoer and Swanepoel 2008). In our experience, the KDE is typically an underestimate in the tails, which can lead to an explosion in the self-density ratio estimate ${{\hat{\text {r}}}}(\phi , \phi ^{*}) = {{\hat{\text {p}}}}_{m}(\phi ) \mathop {/} {{\hat{\text {p}}}}_{m}(\phi ^{*})$. Hence, improbable values for $\phi ^{*}$ are accepted far too often. Once at this improbable value, i.e. when $\phi$ is improbable under $\text {p}_{m}(\phi )$, the error in the KDE then leads to a dramatically reduced value for the acceptance probability. This results in Markov chains that get stuck at improbable values. For example, see the top left panel of Fig. 5.

In which stage this instability arises depends on which prior marginal densities are intractable, and how the terms in Eq. (3) are apportioned across the stages. In the example of Sect. 4, $\text {p}_{1}(\phi )$ is unknown and is part of both stage one (in Eq. (4)) and stage two (via $\text {p}_{\text {pool}}(\phi )$ in Eq. (5)). Thus both stages are numerically brittle. Our second example, contained in Sect. 5, represents a more typical scenario, where the first submodel posterior is used as the proposal for the melded posterior. In this case, all unknown prior marginal terms are factorised into the stage two target, and the instability is confined to the second stage.

## Self-density ratio estimation

As described in Sect. 2.4, the self-density ratios associated with both $\text {p}_{1}(\phi )$ and $\text {p}_{2}(\phi )$ may be required by the two-stage MCMC algorithm. To simplify notation, we consider in this section a generic joint density $\text {p}(\phi , \gamma )$ that we can evaluate pointwise, but whose marginal $\text {p}(\phi ) = \int \text {p}(\phi , \gamma )\text {d}\gamma$ we cannot obtain analytically. Our interest is in the *self-density ratio* evaluated at $\phi _{\text {nu}}$ and $\phi _{\text {de}}$ (the subscripts are abbreviations of numerator and denominator respectively) which we denote as

$$\begin{aligned} \text {r}(\phi _{\text {nu}}, \phi _{\text {de}}) = \frac{ \text {p}(\phi _{\text {nu}}) }{ \text {p}(\phi _{\text {de}}) } = \frac{ \int \text {p}(\phi _{\text {nu}}, \gamma ) \text {d} \gamma }{ \int \text {p}(\phi _{\text {de}}, \gamma ) \text {d} \gamma }. \end{aligned}$$

In our examples we set $\phi _{\text {nu}}= \phi$ and $\phi _{\text {de}}= \phi ^{*}$ for use in Eqs. (4) and (5); and define $\gamma = (\psi _{m}, Y_{m})$ and $\text {p}= \text {p}_{m}$ where $m= 1$ or 2 as appropriate (see Sects. 4 and 5 for details).

To avoid the numerical issues associated with the naive approach, we need to improve the ratio estimate $\widehat{\text {r}}(\phi _{\text {nu}}, \phi _{\text {de}})$ for improbable values of $\phi _{\text {nu}}$ and $\phi _{\text {de}}$, e.g. values more than two standard deviations away from the mean. The fundamental flaw in the naive approach in this context is that it minimises the absolute error in the high density region (HDR) of $\text {p}(\phi )$, i.e. the region $R_{\varepsilon }(\text {p}(\phi )) = \{\phi : \text {p}(\phi ) > \varepsilon \}$. But this is not necessarily the sole region of interest, and we are concerned with minimising the relative error. To address this we reweight $\text {p}(\phi )$ towards a particular region, and thus obtain a more accurate estimate in that region. We then exploit the fact that we only interact with the prior marginal distribution via its self-density ratio to combine estimates from multiple reweighted distributions.

### Single weighting function

We can shift $\text {p}(\phi )$ by multiplying the joint distribution $\text {p}(\phi , \gamma )$ by a known weighting function $\text {w}(\phi ;\xi )$, controlled by parameter $\xi$, then account for this shift in our KDE. This will improve the accuracy of the KDE in the region to which we shift the marginal. We first generate $N$ samples denoted $\{(\phi _{n}, \gamma _{n})\}_{n= 1}^{N}$, from a weighted version of the joint distribution

7 $$\begin{aligned} \{(\phi _{n}, \gamma _{n})\}_{n= 1}^{N} \, \sim \, \frac{1}{Z_{1}} \text {p}(\phi , \gamma ) \text {w}(\phi ; \xi ), \end{aligned}$$

where $Z_{1} = \iint \text {p}(\phi , \gamma ) \text {w}(\phi ; \xi ) \text {d}\phi \text {d}\gamma$ is the normalising constant. The samples $\{\phi _{n}^{(\text {s})}\}_{n= 1}^{N}$, obtained by ignoring the samples of $\gamma _{n}$, are distributed according to a weighted version $\text {s}(\phi ; \xi )$ of the marginal distribution $\text {p}(\phi )$

$$\begin{aligned} \{\phi _{n}^{(\text {s})}\}_{n= 1}^{N} \, \sim \, \frac{1}{Z_{2}} \text {p}(\phi )\text {w}(\phi ; \xi ) = \text {s}(\phi ; \xi ), \end{aligned}$$

where $Z_{2} = \int \text {p}(\phi ) \text {w}(\phi ; \xi ) \text {d}\phi$. Typically (7) cannot be sampled by simple Monte Carlo; instead we employ MCMC.

Using the samples $\{\phi _{n}^{(\text {s})}\}_{n= 1}^{N}$ from $\text {s}(\phi ; \xi )$ we compute a weighted kernel density estimate (Jones 1991), with bandwidth $h$, kernel $\text {K}_{h}$, and normalising constant $Z_{3}$

8 $$\begin{aligned} {\hat{\hat{\text {p}}}}(\phi ) = \frac{1}{Z_{3} Nh} \sum _{n= 1}^{N} (\text {w}(\phi _{n}; \xi ))^{-1} \text {K}_{h}(\phi - \phi _{n}^{(\text {s})}), \end{aligned}$$

and form our weighted-sample self-density ratio estimate

$$\begin{aligned} {\hat{\hat{\text {r}}}}(\phi _{\text {nu}}, \phi _{\text {de}}) = \frac{ {\hat{\hat{\text {p}}}}(\phi _{\text {nu}}) }{ {\hat{\hat{\text {p}}}}(\phi _{\text {de}}) } = \frac{ \sum _{n= 1}^{N} (\text {w}(\phi _{n}; \xi ))^{-1} \text {K}_{h}(\phi _{\text {nu}}- \phi _{n}^{(\text {s})}) }{ \sum _{n= 1}^{N} (\text {w}(\phi _{n}; \xi ))^{-1} \text {K}_{h}(\phi _{\text {de}}- \phi _{n}^{(\text {s})}) } . \end{aligned}$$

The cancellation of the normalisation constant $Z_{3}$ is crucial, as accurately estimating constants like $Z_{3}$ is known to be challenging.

### Choice of weighting function

The choice of $\text {w}(\phi ;\xi )$ affects both the validity and efficacy of our methodology. The weighted marginal $\text {s}(\phi ; \xi )$ must satisfy the requirements for a density for our method to be valid. Hence, the specific form of $\text {w}(\phi ;\xi )$ is subject to some restrictions. Our first requirement is that $\text {w}(\phi ;\xi ) > 0$ for all $\phi$ in the support of $\text {p}(\phi , \gamma )$. We also require that the weighted joint distribution, defined in (7), has finite integral, to ensure that it can be normalised to a probability distribution, and that the marginal $\text {s}(\phi ; \xi )$ is positive over the support of interest, also with finite integral.

### Multiple weighting functions

The methodology of Sect. 3.1 produces a single estimate ${\hat{\hat{\text {r}}}}(\phi _{\text {nu}}, \phi _{\text {de}})$ using ${\hat{\hat{\text {p}}}}(\phi )$ from Eq. (8). It is accurate for values in the HDR of $\text {s}(\phi ; \xi )$, i.e. $R_{\varepsilon }(\text {s}(\phi ; \xi ))$, and we can control the location of $R_{\varepsilon }(\text {s}(\phi ; \xi ))$ through $\xi$. This is similar to importance sampling, with $\text {s}(\phi ; \xi )$ acting as the proposal density. Nakayama (2011) notes importance sampling can be used to improve the mean square error (MSE) of a KDE in a specific local region, at the cost of an increase in global MSE. To ameliorate the decrease in global performance, we specify multiple regions in which we want accurate estimates for ${\hat{\hat{\text {p}}}}(\phi )$, and then combine the corresponding estimates of ${\hat{\hat{\text {r}}}}(\phi _{\text {nu}}, \phi _{\text {de}})$ to provide a single estimate that is accurate across all regions.

We elect to use $W$ different weighting functions, indexed by $w= 1, \ldots , W$, with function-specific parameters $\xi _{w}$ denoted $\text {w}(\phi ;\xi _{w})$. Samples are then drawn from each of the $W$ weighted distributions $\text {s}_{w}(\phi ; \xi _{w}) \propto \text {p}(\phi ) \text {w}(\phi ;\xi _{w})$. Denote the samples from the $w^{th}$ weighted distribution by $\{\phi _{n}^{(\text {s}_{w})}\}_{n= 1}^{N}$. Each set of samples produces a separate ratio estimate ${\hat{\hat{\text {r}}}}_{w}(\phi _{\text {nu}}, \phi _{\text {de}})$ in the manner described in Sect. 3.1.

Each individual ${\hat{\hat{\text {r}}}}_{w}$ is accurate (in terms of relative accuracy) only in the HDR of $\text {s}_{w}(\phi ; \xi _{w})$. Thus, when combining multiple ratio estimates, simply taking the mean of all $w= 1, \ldots , W$ estimates (for a specific value of $\phi _{\text {nu}}$ and $\phi _{\text {de}}$) would not make use of our knowledge about the region in which ${\hat{\hat{\text {r}}}}_{w}$ is accurate. We therefore propose a weighted average of all the individual ratio estimates, where the weights approximately come from $\text {s}_{w}(\phi _{\text {nu}}; \xi _{w}) \text {s}_{w}(\phi _{\text {de}}; \xi _{w})$ – this quantity is largest when ${\hat{\hat{\text {r}}}}_{w}(\phi _{\text {nu}}, \phi _{\text {de}})$ is most accurate. This ensures the more accurate terms are given more weight in our final estimate. Specifically, we use $\{\phi _{n}^{(\text {s}_{w})}\}_{n= 1}^{N}$ to compute a standard KDE of $\text {s}_{w}(\phi ; \xi _{w})$

$$\begin{aligned} {\hat{\text {s}}}_{w}(\phi ; \xi _{w}) = \frac{1}{Nh} \sum _{n= 1}^{N} \text {K}_{h}(\phi - \phi _{n}^{(\text {s}_{w})}). \end{aligned}$$

Finally, we form the weighted-sample self-density ratio estimate ${\hat{\hat{\text {r}}}}_{\text {WSRE}}(\phi _{\text {nu}}, \phi _{\text {de}})$, which is a weighted mean of the individual ratio estimates

$$\begin{aligned} {\hat{\hat{\text {r}}}}_{\text {WSRE}}(\phi _{\text {nu}}, \phi _{\text {de}}) = \frac{1}{Z_{4}} \sum _{w= 1}^{W} {\hat{\text {s}}}_{w}(\phi _{\text {nu}}, \phi _{\text {de}}; \xi _{w}) {\hat{\hat{\text {r}}}}_{w}(\phi _{\text {nu}}, \phi _{\text {de}}), \end{aligned}$$

where $${{\hat{\text {s}}}}_{w} (\phi _{\text {nu}}, \phi _{\text {de}}; \xi _{w}) = {{\hat{\text {s}}}}_{w} (\phi _{\text {nu}}; \xi _{w})\,{{\hat{\text {s}}}}_{w} (\phi _{\text {de}}; \xi _{w})$$ and $$Z_{4} = \sum _{w= 1}^{W} {{\hat{\text {s}}}}_{w}(\phi _{\text {nu}}, \phi _{\text {de}}; \xi _{w})$$.

### Choosing values for $\xi _{w}$

Consider a *D*-dimensional $\phi = (\phi ^{[1]}, \ldots , \phi ^{[D]})$ where $\phi ^{[d]}$ is the $d^{th}$ component of $\phi$, for $d = 1, \ldots , D$. Assume we have a compact region of interest for the $d^{th}$ component denoted $A_{d} = [a_{d}, b_{d}] \subseteq \text {supp}(\phi ^{[d]})$, such that the overall region of interest *A* can be defined as the Cartesian product of component-wise regions of interest . We are interested in accurately evaluating the self-density ratio for two points in this region. We will obtain *W* choices for $\xi _{w}$ by specifying *V* weighting functions for each of the *D* components, such that $W = V^{D}$.

Assume that the weighting function $\text {w}(\phi ; \xi )$ is composed of *D* independent component weighting functions

$$\begin{aligned} \text {w}(\phi ; \xi ) = \prod _{d = 1}^{D} \text {m}(\phi ^{[d]}; \xi ^{[d]}), \end{aligned}$$

where $\xi ^{[d]}$ is the $d^{th}$ component of $\xi$. We can then define the marginal of the weighted target

$$\begin{aligned} \text {t}(\phi ^{[d]}; \xi ^{[d]}) = \int \text {s}(\phi ; \xi ) \text {d}\phi ^{[-d]}, \end{aligned}$$

where $\phi ^{[-d]}$ represents the $D - 1$ components of $\phi$ that are not $\phi ^{[d]}$. For typical choices of $\xi$ and $\text {w}(\phi ; \xi )$, the corresponding HDR of $\text {t}(\phi ^{[d]}; \xi ^{[d]})$ does not span the region of interest. That is, $|R_{\varepsilon }(\text {t}(\phi ^{[d]}; \xi ^{[d]})) |\ll |A_{d} |$.

Our aim is to choose, for each of the *d* components, values $v = 1, \ldots , V$ of $\xi ^{[d]}$ denoted $\{\xi _{v, d}\}_{v = 1}^{V}$, yielding weighting functions $\text {m}(\phi ^{[d]}, \xi _{v, d})$ and corresponding $\text {t}(\phi ^{[d]}, \xi _{v, d})$, such that $\bigcup _{v = 1}^{V} R_{\varepsilon }(\text {t}(\phi ^{[d]}; \xi _{v, d})) \approx A_{d} = [a_{d}, b_{d}]$. We employ the following heuristic argument, first choosing a “minimum” $\xi _{1, d}$ and a “maximum” $\xi _{V, d}$ such that

$$\begin{aligned} \begin{aligned} \xi _{1, d} :\, a_{d} \in R_{\varepsilon }(\text {t}(\phi ^{[d]}; \xi _{1, d})), \\ \xi _{V, d} :\, b_{d} \in R_{\varepsilon }(\text {t}(\phi ^{[d]}; \xi _{V, d})). \end{aligned} \end{aligned}$$

In words, we choose a minimum value $\xi _{1, d}$ so that the corresponding HDR of the weighted target includes the lower limit of the region of interest. An analogous argument is used to choose the maximum $\xi _{V, d}$. We then interpolate $V - 2$ values between $\xi _{1, d}$ and $\xi _{V, d}$ ensuring that there is sufficient, but not complete, overlap between the corresponding HDRs.

Denote an element from the set of all *W* possible values for the parameter of the weighting function with , noting that $\xi _{w}$ is a *D*-vector.

The practitioner typically has some knowledge of $p(\phi )$ and *A* from prior predictive checks and previous attempts at running the two-stage sampler. Thus only a small number of trial-and-error attempts should be needed to determine $\xi _{1, d}$ and $\xi _{V, d}$ for all dimensions. These attempts are also used to check for overlap between the HDRs, and increase *V* if the overlap is insufficient. Section 6 contains further discussion of this selection process and its relationship to umbrella sampling (Torrie and Valleau 1977)

### Practicalities and software

In our examples we use Gaussian density functions for $\text {m}(\phi ^{[d]};\xi _{v, d})$,

$$\begin{aligned} \text {m}(\phi ^{[d]};\xi _{v, d}) = \frac{1}{\sqrt{2 \pi \sigma ^{2}_{v, d}}} \text {exp} \left\{ - \frac{1}{2 \sigma ^{2}_{v, d}} (\phi - \mu _{v, d})^2 \right\} , \end{aligned}$$

with $\xi _{v, d} = (\mu _{v, d}, \sigma ^{2}_{v, d})$, though we fix $\sigma ^{2}_{v, d} = \sigma ^{2}_{d}$ for all *v*. Our definition of sufficient overlap is that 0.95 empirical quantile of $\text {t}(\phi ^{[d]}; \xi _{v, d})$ is equal or slightly greater than the 0.05 empirical quantile of $\text {t}(\phi ^{[d]}; \xi _{v + 1, d})$, for $v = 1, \ldots , V - 1$.

Our implementation of our WSRE methodology is available in an R (R Core Team 2021) package at https://github.com/hhau/wsre. It is built on top of Stan (Carpenter et al. 2017) and Rcpp (Eddelbuettel and François 2011). Package users supply a joint density $\text {p}(\phi , \gamma )$ in the form of a Stan model; choose the parameters $\xi _{w}$ of each of the $W$ weighting functions; and the number of samples $N$ drawn from each $\text {s}_{w}(\phi ; \xi _{w})$. The combined estimate ${\hat{\hat{\text {r}}}}_{\text {WSRE}}(\phi _{\text {nu}}, \phi _{\text {de}})$ is returned. A vignette on using wsre is included in the package, and documents the specific form of Stan model required.

## An evidence synthesis for estimating the efficacy of HIV screening

To illustrate our approach we artificially split an existing joint model into two submodels, then compare the melded posterior estimates obtained by the two-stage algorithm using the naive and WSRE approaches. Artificially splitting this joint model serves several purposes: it demonstrates that the numerical instability can occur in a simple, low dimensional setting; we can obtain a good parametric approximation to the prior marginal to use as a reference; and the simplicity of the model allows us to focus on our methodology, not the complexity of the model.

### Model

The model is a simple evidence synthesis model for inferring the efficacy of HIV screening in prenatal clinics (Ades and Cliffe 2002), and has 8 *basic parameters* $\rho _{1}, \rho _{2}, \ldots , \rho _{8}$, which are group membership probabilities for particular risk groups and subgroups thereof. The first risk group partitions the prenatal clinic attendees into those born in sub-Saharan Africa (SSA), injecting drug users (IDU), and the remaining women. These groups have corresponding membership probabilities $\rho _{1}, \rho _{2}$, and $1 - \rho _{1} - \rho _{2}$. The groups are subdivided based on whether they are infected with HIV, with group specific HIV positivity $\rho _{3}, \rho _{4}$ and $\rho _{5}$ respectively; and if they had already been diagnosed before visiting the clinic, with pre-clinical diagnosis probabilities $\rho _{6}, \rho _{7}$ and $\rho _{8}$. An additional probability is also included in the model, denoted $\rho _{9}$, which considers the prevalence of HIV serotype B. This parameter enables the inclusion of study 12, which further informs the other basic parameters. Table 1 summarises the full joint model, including the $s = 1, \ldots , 12$ studies with observations $y_{s}$ and sample size $n_{s}$; the basic parameters $\rho _{1}, \ldots , \rho _{9}$; and the link functions that relate the study proportions $\pi _{1}, \ldots , \pi _{12}$ to the basic parameters.

**Table 1:** HIV example: Study probabilities, their relationships to the basic parameters, and data used to inform the probabilities. For full details on the sources of the data see Ades and Cliffe (2002).

Parameter	Data		
	*y*	*n*	*y*/*n*
$\pi _1 = \rho _{1}$	11,044	104,577	0.106
$\pi _2 = \rho _{2}$	12	882	0.014
$\pi _3 = \rho _{3}$	252	15,428	0.016
$\pi _4 = \rho _{4}$	10	473	0.021
$\pi _5 = \frac{\rho _{4}\rho _{2} + \rho _{5}(1-\rho _{1} -\rho _{2})}{1-\rho _{1} }$	74	136,139	0.001
$\pi _6 = \rho _{3}\rho _{1} + \rho _{4}\rho _{2} + \rho _{5}(1-\rho _{1} -\rho _{2})$	254	102,287	0.002
$\pi _7 = \frac{\rho _{6}\rho _{3}\rho _{1} }{\rho _{6}\rho _{3}\rho _{1} + \rho _{7}\rho _{4}\rho _{2} + \rho _{8}\rho _{5}(1-\rho _{1} -\rho _{2})}$	43	60	0.717
$\pi _8 = \frac{\rho _{7}\rho _{4}\rho _{2}}{\rho _{7}\rho _{4}\rho _{2} + \rho _{8}\rho _{5}(1-\rho _{1} -\rho _{2})}$	4	17	0.235
$\pi _9 = \frac{\rho _{6}\rho _{3}\rho _{1} +\rho _{7}\rho _{4}\rho _{2}+\rho _{8}\rho _{5}(1-\rho _{1} -\rho _{2})}{\rho _{3}\rho _{1} + \rho _{4}\rho _{2} + \rho _{5}(1-\rho _{1} -\rho _{2})}$	87	254	0.343
$\pi _{10} = \rho _{7}$	12	15	0.800
$\pi _{11} = \rho _{9}$	14	118	0.119
$\pi _{12} = \frac{\rho _{4}\rho _{2} + \rho _{9}\rho _{5}(1-\rho _{1} -\rho _{2})}{\rho _{4}\rho _{2} + \rho _{5}(1-\rho _{1} -\rho _{2})}$	5	31	0.161

We make one small modification to original model of Ades and Cliffe (2002), to better highlight the impact of WSRE on the melded posterior estimate. The original model adopts a flat, Beta(1, 1) prior for $\rho _{9}$. This induces a prior on $\pi _{12}$ that is not flat, but not overly informative, making it difficult to demonstrate the issues caused by an inaccurate density estimate of the tail of the prior marginal distribution. Instead, we adopt a Beta(3, 1) prior for $\rho _{9}$. This prior would have been reasonable for the time and place in which the original evidence synthesis was constructed, since the distribution of HIV serotypes differs considerably between North America and sub-Saharan Africa (Hemelaar 2012).

The code to reproduce this example is available at https://github.com/hhau/presanis-conflict-hiv-example.

### Submodels formed by splitting

In the full joint model study 12 informs the probability $\pi _{12}$, and provides indirect evidence for the basic parameters through the deterministic link function

$$\begin{aligned} \pi _{12} = \frac{ \rho _{2}\rho _{4} + \rho _{9}\rho _{5}(1 - \rho _{1} - \rho _{2}) }{ \rho _{2}\rho _{4} + \rho _{5}(1 - \rho _{1} - \rho _{2}) }. \end{aligned}$$

Figure 1 is a DAG of the basic parameters in the full model that relate to $\pi _{12}$. We consider splitting the model at the node corresponding to the expected proportion $\pi _{12}$ in study 12, i.e. we set the common quantity $\phi = \pi _{12}$.

The first submodel ($m= 1$) considers data from studies 1 to 11 $Y_{1} = (y_{1}, \ldots , y_{11})$, corresponding study proportions $(\pi _{1}, \ldots , \pi _{11})$, and all basic parameters $\psi _{1} = (\rho _{1}, \ldots , \rho _{9})$. Note that the study proportions are implicitly defined because they are deterministic functions of the basic parameters. The joint distribution of this submodel is

$$\begin{aligned} \text {p}_{1} (\rho _{1}, \ldots , \rho _{9}, y_{1}, \ldots , y_{12}) = \text {p}(\rho _{1}) \ldots \text {p}(\rho _{9}) \prod _{s = 1}^{11} \text {p}(y_{s} \mid \pi _{s}). \end{aligned}$$

The prior $\text {p}_{1}(\pi _{12})$ on the common quantity $\phi = \pi _{12}$ is implicitly defined, so its analytic form is unknown, hence it needs to be estimated.

The second submodel ($m= 2$) pertains specifically to study 12, with data $Y_{2} = y_{12}$, the study 12 specific probability $\phi = \pi _{12}$, and $\psi _{1} = \varnothing$. The joint distribution is $\text {p}_{2}(\pi _{12}, y_{12}) = \text {p}_{2}(\pi _{12}) \text {p}(y_{12} \mid \pi _{12}).$ In more complex examples $\text {p}_{2}(\phi )$ may be implicitly defined, and contribute substantially to the melded posterior. However, in this simple example we are free to choose $\text {p}_{2}(\pi _{12}) = \text {p}_{2}(\phi )$, and opt for a Beta(1, 1) prior.

### Self-density ratio estimation

We now compute the self-density ratio estimate ${\hat{\hat{\text {r}}}}_{\text {WSRE}}(\phi _{\text {nu}}, \phi _{\text {de}})$ of $\text {p}_{1}(\phi _{\text {nu}}) \mathop {/} \text {p}_{1}(\phi _{\text {de}})$. In the notation defined in Sect. 3.4, this example has $D = 1$, and we use $V = W = 7$ Gaussian weighting functions. We fix the variance parameter of the weighting function $\sigma _{w}^{2} = 0.08^2$ for all $w$, and use the heuristic described in Sect. 3.4 to choose values for the mean parameter of the weighting function. Specifically, we set the minimum to be $\xi _{1, 1} = \mu _{1} = 0.05$, with maximum $\xi _{7, 1} = \mu _{7} = 0.08$ and 5 additional, equally spaced values between these extrema. We draw 3000 MCMC samples in total, apportioned equally across the 7 weighting functions. We thus draw 428 post warmup MCMC samples from each weighted target.

**Fig. 2:**
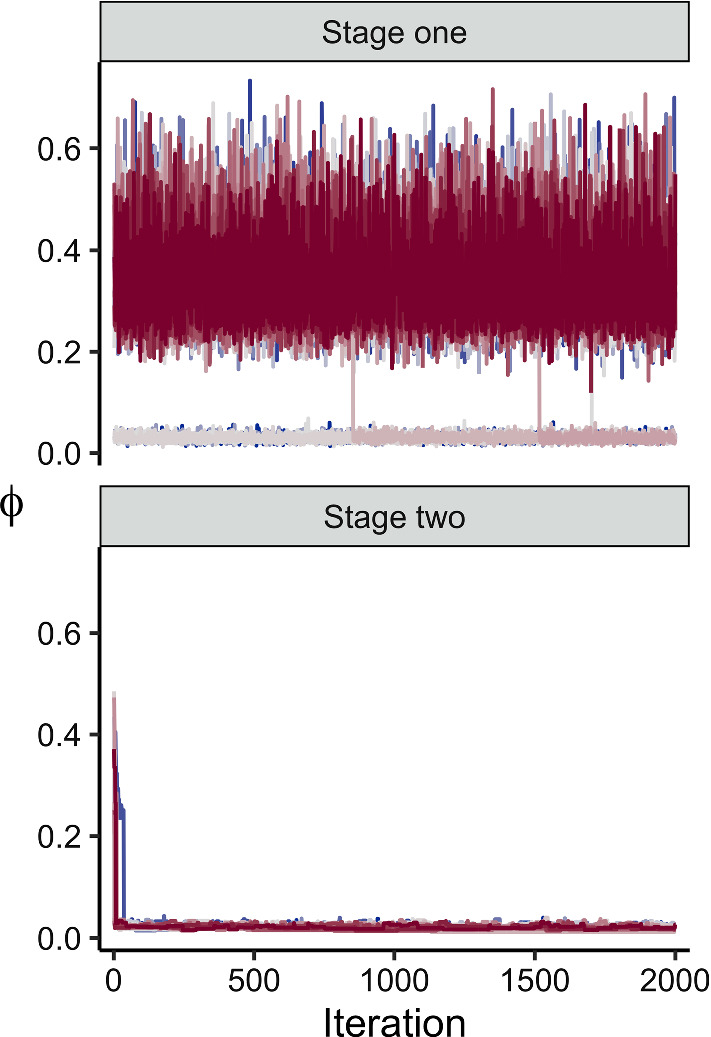
*Top*: Stage one trace plot for $\phi$ using the naive method. At any moment in time chains can jump to the spurious mode, which is an artefact of ${{\hat{\text {p}}}}_{1}(\phi )$. *Bottom*: Corresponding stage two trace plot. The stage two target has the same numerical instability, and because the stage one samples are the proposal distribution, all chains encounter the instability

**Fig. 3:**
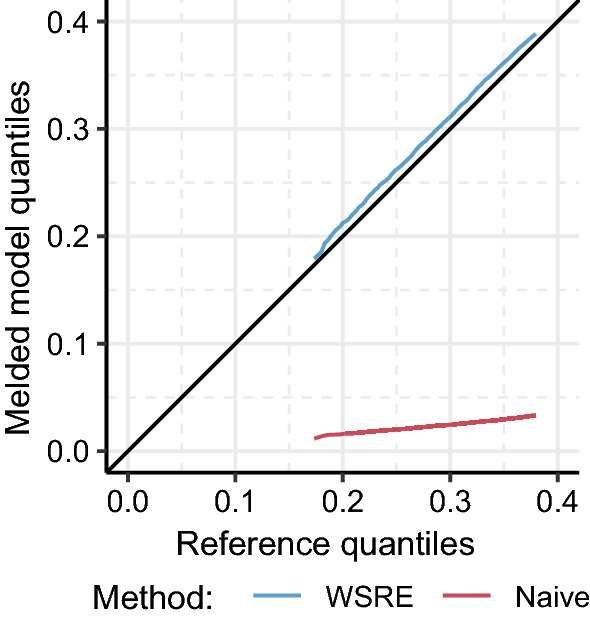
Quantile-quantile plot of the melded posterior quantiles using the WSRE approach (blue) and the naive approach (red). Both methods are comparable to the quantiles from the reference sample (x-axis)

### Results

We compare the melded posterior obtained by the naive approach and using WSRE. For a fair comparison, we estimate the prior marginal distribution of interest ${\widehat{\text {p}}}_{1}(\phi )$ using 3000 Monte Carlo samples, This set-up is slightly advantageous for the naive approach, which uses Monte Carlo samples, rather than the MCMC samples of the self-density ratio estimate; the naive approach makes use of a sample comprised of 3000 effective samples, whilst the self-density ratio estimate uses fewerthan 3000 effective samples. A reference estimate of the melded posterior is obtained using a parametric density estimate ${{\hat{\text {p}}}}_{\text {ref}, 1}(\phi )$ for the unknown prior marginal, based on $5 \times 10^{6}$ prior samples. The reference sample also contains some error, as ${{\hat{\text {p}}}}_{\text {ref}, 1}(\phi )$ is not perfect. However, in the absence of an analytic form for $\text {p}_{1}(\phi )$ it serves as a very close approximation. We estimate the melded posterior using the two-stage sampler of Sect. 2.2, targeting in stage one

9 $$\begin{aligned} \text {p}_{\text {meld}, 1} (\phi , \psi _{1} \mid Y_{1}) \propto \frac{ \text {p}_{1}(\phi , \psi _{1}, Y_{1}) }{ \text {p}_{1}(\phi ) }. \end{aligned}$$

and the full melded posterior in stage two. To demonstrate the numerical instability of interest, we run 24 chains that target $\text {p}_{\text {meld}, 1}$ in (9) using the naive approach.

The top panel of Fig. 2 displays the trace plot of the post-warmup samples. Many chains have already converged to a spurious model around $\phi \approx 0.02$, and other chains jump to this mode after a variable number of additional iterations. As discussed in Sect. 2.4, this mode is an artefact of the naive KDE employed for ${{\hat{\text {p}}}}_{1}(\phi )$, and is also visible in the corresponding stage two trace plot (bottom panel of Fig. 2). Because the stage one samples act as the proposal for stage two, all stage two chains quickly jump to the spurious mode.

The samples surrounding the spurious mode introduce substantial bias in estimate of the melded posterior under the naive approach. This is visible in the quantile-quantile plot in Fig. 3, where the naive approach produces an implausible estimate compared to the reference quantiles. In contrast, the WSRE approach rectifies the numerical instability, and uses the two-stage sampler to produce a sensible estimate of the melded posterior.

## An evidence synthesis to estimate the severity of the H1N1 pandemic

We now consider a more involved example, where the prior for the common quantity does not have an analytical form under either submodel, and the two priors contain a substantially different quantity of information. Presanis et al. (2014) undertook a large evidence synthesis in order to estimate the severity of the H1N1 pandemic amongst the population of England. This model combines independent data on the number of suspected influenza cases in hospital’s intensive care unit (ICU) into a large severity model. Here, we reanalyse the model introduced in Goudie et al. (2019) that uses Markov melding to join the independent ICU model ($m= 1$) with a simplified version of the larger, complex severity model ($m= 2$). In this example the melded model has no obvious implied joint model, so there are no simple “gold standard” joint model estimates to use as a baseline reference. However, we demonstrate that the naive approach is highly unstable, whereas the WSRE approach produces stable results. The code to reproduce all figures and outputs for this example is available at https://github.com/hhau/full-melding-example.

### ICU submodel

The data for the ICU submodel ($m= 1$) are aggregate weekly counts of patients in the ICU of all the hospitals in England, for 78 days between December 2010 and February 2011. Observations were recorded of the number of children $a = 1$ and adults $a = 2$ in the ICU on days $U = \{8, 15, \ldots , 78\}$, and we denote a specific weekly observation as $y_{a, t}$ for $t \in U$.

To appropriately model the temporal nature of the weekly ICU data we use a time inhomogeneous, thinned Poisson process with rate parameter $\lambda _{a, t}$ for $t \in T$ where $T = \{1, 2, \ldots , 78\}$. This is the expected number of new ICU admissions; the corresponding age group specific ICU exit rate is $\mu _{a}$. There is also a discrepancy between the observation times $U$ and the daily support of our Poisson process $T$. We address this in the observation model

10 $$\begin{aligned} y_{a, t}&\sim \text {Pois}(\eta _{a, t}), \,\, t \in U, \end{aligned}$$

11 $$\begin{aligned} \eta _{a, t}&= \sum _{u = 1}^{t} \lambda _{a, u} \exp \{-\mu _{a}(t - u)\}, \,\, t \in T, \end{aligned}$$

through different supports for $t$ in Eqs. (10) and (11). An identifiability assumption of $\eta _{a, 1} = 0$ is required, which enforces the reasonable assumption that no H1N1 influenza patients were in the ICU at time $t~=~0$.

Weekly virological positivity data are available at weeks $V = \{1, \ldots , 11\}$, and inform the proportion of influenza cases which are attributable to the H1N1 virus $\pi _{a, t}^{\text {pos}}$. The virology data consists of the number of H1N1-positive swabs $z_{a, v}^{\text {pos}}$ and the total number of swabs $n_{a, z}^{\text {pos}}$ tested for influenza that week. This proportion relates the counts to $\pi _{a, t}^{\text {pos}}$ via a truncated uniform prior on $\pi _{a, t}^{\text {pos}}$,

$$\begin{aligned} \pi _{a, t}^{\text {pos}}&\sim \text {Unif}(\omega _{a, v}, 1), \,\, t \in T \,\, \\ z_{a, v}^{\text {pos}}&\sim \text {Bin}(n_{a, z}^{\text {pos}}, \omega _{a, v}) , \,\, v \in V,\ \end{aligned}$$

with $v = 1$ for $t = 1, 2, \ldots , 14$, and $v = \lfloor (t - 1) \mathop {/} 7 \rfloor$ for $t = 15, 16, \ldots , 78$ to align the temporal indices. The positivity proportion $\pi _{a, t}^{\text {pos}}$ is combined with $\lambda _{a, t}$ to compute the lower bound on the total number of H1N1 cases $\phi _{a} = \sum \limits _{t \in T} \pi _{a, t}^{\text {pos}} \lambda _{a,t}$ where $\phi = (\phi _{1}, \phi _2)$ is the quantity common to both submodels. This summation is a non-invertible function, which necessitates either considering this model in stage one of our two-stage sampler, or appropriately augmenting the definition of $\phi _{a}$ such that it is invertible. We elect to consider this submodel in stage one, and further discuss model ordering in Sect. 6.

Lastly, we specify priors for the remaining parameters. A lognormal random-walk is used for the expected number of new admissions

$$\begin{aligned} \log (\lambda _{a, 1}) {\sim }\text {Unif}(0, 250), \,&\, \log (\lambda _{a, t}) \sim \text {N}(\log (\lambda _{a, t - 1}), \nu _{a}^{-2}), \\ \nu _{a}&\sim \text {Unif}(0.1, 2.7), \end{aligned}$$

for $t = 2, 3, \ldots , 78$ and $a = {1, 2}$. Age group specific exit rates have informative priors (Presanis et al. 2014)

$$\begin{aligned} \begin{aligned}&\mu _{1} = \exp \{-\alpha \}, \,\, \mu _{2} = \exp \{-(\alpha + \beta )\}, \\&\alpha \sim \text {N}(2.7058, 0.0788^2), \,\, \beta \sim \text {N}(-0.4969, 0.2048^2), \end{aligned} \end{aligned}$$

and the lower bound on the positivity proportion has a flat prior, $\omega _{a, v} \sim \text {Unif}(0, 1)$, for $v \in V$.

### Severity submodel

A simplified version of the large severity model ($m= 2$) of Presanis et al. (2014) is considered here, in which parts of the severity model are collapsed into informative priors. The cumulative number of ICU admissions $\phi _{a}$ is assumed to be an underestimate of the true number of ICU admissions due to H1N1, $\chi _{a}$. This motivates

$$\begin{aligned} \phi _{a} \sim \text {Bin}(\chi _{a}, \pi ^{\text {det}}),&\,\, \pi ^{\text {det}} \sim \text {Beta}(6, 4), \\ \chi _{1} \sim \text {LN}(4.93, 0.17^2),&\,\, \chi _{2} \sim \text {LN}(7.71, 0.23^2), \end{aligned}$$

where $\pi ^{\text {det}}$ is the age group constant probability of detection, and the priors on $\chi _{a}$ appropriately summarise the remainder of the large severity model.

### Prior distributions, stage one target

**Fig. 4:**
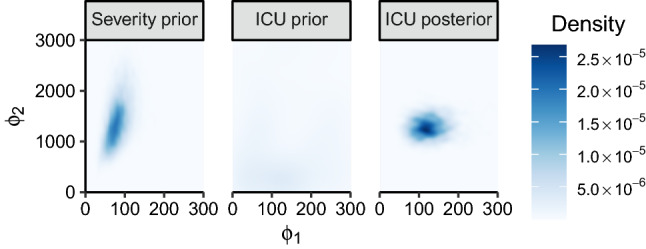
Heatmap of the severity submodel prior $\text {p}_{2}(\phi )$, ICU submodel prior $\text {p}_{1}(\phi )$, and the stage one (ICU submodel) posterior $\text {p}_{1}(\phi \mid Y_{1})$

Figure 4 displays $\text {p}_{m}(\phi )$ for both submodels, as well as the subposterior for the ICU ($m= 1$) submodel $\text {p}_{1}(\phi \mid Y_{1})$. The melded posterior will be largely influenced by the product of $\text {p}_{1}(\phi \mid Y_{1})$ and $\text {p}_{2}(\phi )$, since $\text {p}_{1}(\phi )$ is effectively uniform (see the centre panel of Fig. 4), and there are no data observed in the severity submodel, i.e. $Y_{2} = \varnothing$. In stage one we target the ICU submodel posterior $\text {p}_{1}(\phi , \psi _{1} \mid Y_{1})$, enabling the use of the original JAGS (Plummer 2018) implementation. These samples for $\phi$ are displayed in the right panel of Fig. 4, and we see that whilst there is substantial overlap with $\text {p}_{2}(\phi )$ (left panel), $\text {p}_{1}(\phi \mid Y_{1})$ is more disperse, particularly for $\phi _{1}$. Our region of interest is thus the HDR of $\text {p}_{1}(\phi \mid Y_{1})$, as the two-stage sampler involves evaluating the samples from $\text {p}_{1}(\phi \mid Y_{1})$ under $\text {p}_{2}(\phi )$.

### Self-density ratio estimation

The stage two acceptance probability for a move from $\phi \rightarrow \phi ^{*}$ where $\phi ^{*} \sim \text {p}_{1}(\phi , \psi _{1} \mid Y_{1})$ is

12 $$\begin{aligned} \alpha (\phi ^{*}, \phi ) = \frac{ \text {p}_{\text {pool}}(\phi ^{*}) \text {p}_{2}(\phi ^{*}, \psi _{2}, Y_{2}) \text {p}_{1}(\phi ) \text {p}_{2}(\phi ) }{ \text {p}_{\text {pool}}(\phi ) \text {p}_{2}(\phi , \psi _{2}, Y_{2}) \text {p}_{1}(\phi ^{*}) \text {p}_{2}(\phi ^{*}) }. \end{aligned}$$

In both the severity and ICU submodels, the prior marginal distribution $\text {p}_{m}(\phi )$ is unknown. This necessitates estimating the self-density ratio for both $\text {p}_{1}(\phi )$ and $\text {p}_{2}(\phi )$. However, the uniformity of $\text {p}_{1}(\phi )$ corresponds to a self-density ratio that is effectively $1$ everywhere. In contrast, the severity submodel prior marginal $\text {p}_{2}(\phi )$ is clearly not uniform over our region of interest; appropriately estimating the melded posterior thus requires an accurate estimate of $\text {p}_{2}(\phi ) \mathop {/} \text {p}_{2}(\phi ^{*})$.

To obtain the WSRE estimate of $\text {p}_{2}(\phi ) \mathop {/} \text {p}_{2}(\phi ^{*})$ we first note that, in the notation of Section 3.4, $\phi = (\phi _{1}, \phi _{2}) = (\phi ^{[1]}, \phi ^{[2]})$, thus $D = 2$. We set $V = 10$, resulting in $W = 10^{2}$ weighting functions, and we draw $10^{3}$ samples from each weighted target for a total of $10^{5}$ MCMC samples. The Gaussian weighting functions for the first component $\text {m}(\phi ^{[1]}; \xi _{v, 1})$ have the variance parameter fixed to $25^{2}$; for the mean parameter we set $\xi _{1, 1} = 30$, $\xi _{V, 1} = 275$, with 8 other equally spaced values between these extrema. For $\text {m}(\phi ^{[2]}; \xi _{v, 2})$ we set the variance parameter to $250^2$, with $\xi _{1, 2} = 500$, $\xi _{V, 2} = 3000$, and interpolate 8 equally spaced values between the extrema. The values for the variance parameters are based on the empirical variance of the samples in Fig. 4.

### Results

**Fig. 5:**
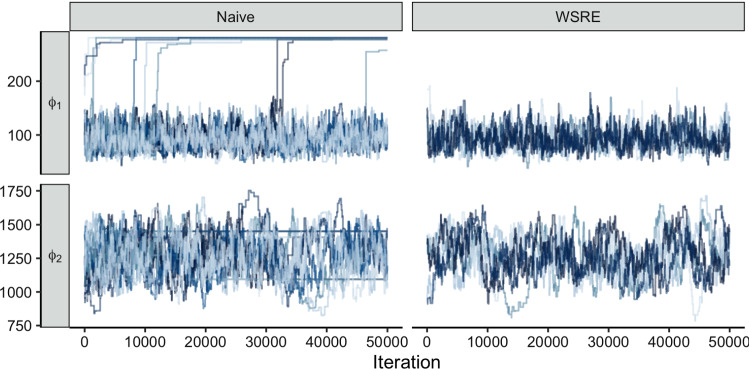
Trace plots of 15 replicate stage two chains for $\phi _{1}$ and $\phi _{2}$, using the naive approach (left column) and the WSRE approach (right column)

We compare the melded posterior estimate obtained using WSRE against the naive approach. For the latter we draw $10^5$ samples from $\text {p}_{2}(\phi )$ so that both approaches have the same number of samples, although the naive approach has a larger effective sample size. Figure 5 displays trace plots of 15 stage two MCMC chains, where $\alpha (\phi ^{*}, \phi )$ is computed using the naive approach (left column), and using WSRE (right column). The erroneous behaviour displayed in the left column is due to underestimation of the tails of ${\widehat{\text {p}}}_{2}(\phi )$ using a standard KDE. This underestimation results in an overestimation of the acceptance probability for proposals in the tails of ${\widehat{\text {p}}}_{2}(\phi )$, since the proposal term ${\widehat{\text {p}}}_{2}(\phi ^{*})$ is in the denominator of Eq. (12). Hence, moves to improbable values of $\phi ^{*}$ have acceptance probabilities that are dominated by Monte Carlo error. Once at this improbable value the error then has the opposite effect; the underestimate yields chains unable to move back to probable values. This produces the monotonic step-like behaviour seen in the top left panel of Fig. 5. Although this behaviour is not visible in all 15 chains, it will eventually occur if the chains are run for more iterations, as a sufficiently improbable value for $\phi ^{*}$ will be proposed. The results from this sampler are thus unstable.

Whilst there is no baseline “truth” to compare to in this example, the sampler that employs ${\hat{\hat{\text {r}}}}_{\text {WSRE}}(\phi , \phi ^{*})$ as an estimate of $\text {p}_{2}(\phi ) \mathop {/} \text {p}_{2}(\phi ^{*})$ produces plausible results, in contrast to the naive approach. No step-like behaviour is visible when employing the WSRE approach (right column of  5). Whilst the between-chain mixing is not optimal, this can be ameliorated by running the chains for longer, which cannot be said for the naive method. This improved behaviour is obtained using the same number of samples from the prior marginal distribution, or weighted versions thereof. Users of this algorithm can be much more confident that the results are not artefactual.

## Discussion

The complexity of many phenomena necessitates intricate, large models. Markov melding allows the practitioner to channel modelling efforts into smaller, simpler submodels, each of which may have data associated with it, then coherently combine these smaller models and disparate data. Multi-stage, sequential sampling methods, such as the sampler used for Markov melding, are important tools for estimating these models in a pragmatic, computationally feasible manner.

In particular, when an analytic form of the prior marginal distribution is not available, we have demonstrated that the two-stage sampling process is particularly sensitive to the corresponding KDE in regions of low probability. Tail probability estimation is an important and recurrent challenge in statistics (Hill 1975; Béranger et al. 2019). We addressed this issue in the Markov melding context by noting that we can limit our focus to the self-density ratio estimate, and sample weighted distributions to improve performance in low probability areas, for lower computational cost than simple Monte Carlo. Our examples show that for equivalent sample sizes, we improve the estimation of the melded posterior compared to the naive approach.

The issue addressed in this paper arises to due differences in the intermediary distributions of the two-stage sampling process, particularly where the proposal distribution is wider than the target distribution. The presence or absence of this issue is dependent upon the order in which the components of the melded model are considered in the sampling process, which is often constrained by the link function used to define $\phi$ in each model. In both our examples the link function is non-invertible. Goudie et al. (2019) show extensions of the link function that render it invertible are valid; that is, the model is theoretically invariant to the choice of extension. However, the practical performance of the two-stage sampler is heavily dependent on the appropriateness of such extensions, and designing such extensions is extremely challenging. Hence, the ordering of the submodels in the two-stage sampler is often predetermined; we are practically constrained by the non-invertible link function. In our examples this corresponds to sampling the less informative model for $\phi$ first. If we are free to choose the ordering of the two-stage sampler, we may still prefer to sample the wider model first, as the melded posterior is more likely to be captured in a reweighted sample from a wider distribution than such a sample from a narrow distribution. However, if the melded posterior distribution is substantially narrower than the stage-one target distribution then we are susceptible to the sample degeneracy and impoverishment problem (Li et al. 2014). Addressing this issue in the melding context, whilst retaining the computational advantages of the two-stage sampler, is an avenue for future work.

The examples we consider contain 1 or 2 dimensional $\phi$. For higher dimensional $\phi$ we anticipate encountering issues associated with the curse of dimensionality. Specifically, the decrease in accuracy of any KDE and increase in the required number of weighting functions will scale exponentially with dimension. Applying the argument in Sect. 3.4 to locate these additional weighting functions will be challenging. As such we recommend WSRE, like other KDE methods, for settings where $\text {dim}(\phi ) \le 5$ (Wand and Jones 1995). This requirement may be relaxed when there is structure in $\phi$ that allows it to be split into lower-dimensional components, such as when $\phi$ contains a collection of subject-specific parameters that are independent a priori. More generally, in high dimensions almost everywhere is a ‘region of low probability’ and the performance of KDEs is known to be poor, making choosing both an appropriate number of weighting functions and their parameters difficult. Machine learning methods have proven to be effective for estimating densities of moderate to high dimension (see Wang and Scott (2019) for a review), however the performance of these methods in low probability regions has not, to our knowledge, been thoroughly investigated.

There are potential alternatives to our weighted-sample self-density ratio estimation technique. Umbrella sampling (Torrie and Valleau 1977; Matthews et al. 2018) aims to accurately estimate the tails of a density $\text {p}(\phi )$ by constructing an estimate ${\widehat{\text {p}}}(\phi )$ from $W$ sets of weighted samples $\{\phi _{n, w} \}_{n= 1}^{N} \sim \text {s}_{w}(\phi ; \xi _{w})$, However, umbrella sampling requires estimates of the normalising constants $Z_{2, w} = \int \text {s}_{w}(\phi ; \xi _{w}) \text {d}\phi$ to combine the density estimates computed from each weighted sample. Our approach is able to avoid computing normalising constants by focusing on the self-density ratio. Umbrella sampling also requires choosing the location of the weighting functions, i.e. choosing $\xi _{w}$ appropriately. A heuristic strategy, similar to that of Sect. 3.4, is seen as necessary by Torrie and Valleau (1977). Adaptive procedures that automatically choose values of $\xi _{w}$ based on other criteria exist, but these assume that $\text {s}_{w}(\phi )$ is a Gaussian distribution (Mitsuta et al. 2018) or operate on a predefined grid of possible values (Wojtas-Niziurski et al. 2013). We cannot use the generic tempering methodology advocated by Matthews et al. (2018), as sampling from $\text {p}(\phi , \gamma )^{1 \mathop {/} \tau }$, for $\tau > 1$, does not generally produce marginal samples from $\text {p}(\phi )^{1 \mathop {/} \tau }$.

Another possibility would be to sample $\text {p}_{\text {meld}}$ using a pseudo-marginal approach (Andrieu and Roberts 2009). A necessary condition of the pseudo-marginal approach is that we possess an unbiased estimate of the target distribution. Kernel density estimation produces biased estimates of $\text {p}(\phi )$ for finite $N$. A KDE can be debiased (Calonico et al. 2018; Cheng and Chen 2019), but doing so requires substantial computational effort. Moreover, we also require an unbiased estimate of $1 \mathop {/} \text {p}(\phi )$. Debiasing estimates of $1 \mathop {/} \text {p}(\phi )$ is possible with pseudo-marginal methods like Russian roulette (Lyne et al. 2015), but Park and Haran (2018) observe prohibitive computational costs when doing so. The presence of both $\text {p}_{\text {pool}}(\phi )$ and $1 \mathop {/} \text {p}(\phi )$ in the melded posterior further complicates the production of an unbiased estimate, particularly when $\text {p}_{\text {pool}}(\phi )$ is formed via logarithmic pooling.
